# Prevalence of Hypertension among High School Students in a Middle Anatolian Province of Turkey

**Published:** 2008-03

**Authors:** Naim Nur, Selma Çetinkaya, Abdülkerim Yilmaz, Adnan Ayvaz, Mustafa Orhan Bulut, Haldun Sümer

**Affiliations:** 1 Department of Public Health; 2 Department of Internal Medicine; 3 Department of Pediatric Diseases, Faculty of Medicine, Cumhuriyet University, 58140-Sivas, Turkey

**Keywords:** Cross-sectional studies, Epidemiology, Hypertension, Prevalence, Risk factors, Turkey

## Abstract

Hypertension may lead to irreversible damages in vital organs, such as heart, brain, and kidney, and may cause death in children if treatments are not given despite early diagnosis. This cross-sectional epidemiological study was conducted during 1 January–31 March 2004 to investigate the prevalence of hypertension among high school students. The study cohort included 1,041 students of six high schools, who were selected from among 14,789 students of 26 high schools in Sivas province of Turkey, using the cluster-sampling method. A questionnaire was used for collecting information from students on age, gender, smoking, and whether they or their families have any diseases. Blood pressure, height, and weight of the participitants were determined by the research group. Students whose repeated systolic or diastolic blood pressures were higher than the 95th percentile were considered to be hypertensive patients. Hypertension was prevalent among 4.4% (n=45) of the students. There was a significant correlation between prevalence of hypertension and body mass index. No significant correlation was found between prevalence of hypertension and other variables, such as smoking, age, gender, and family history of diabetes. The results suggest that hypertension is an important public-health problem among high school students. The results also showed that the body mass index was an important parameter in hypertension in such a study group. Researchers should consider overweight a causative risk factor for development of hypertension in early-onset groups.

## INTRODUCTION

The incidence of hypertension in childhood varies from 1% to 3% (1,2). Hypertension, a chronic disease, can lead to failure in vital organs, such as heart, brain, and kidney, and can cause death. Unlike adults, the incidence of essential (primary) hypertension among children is lower, and 75–80% of all childhood hypertension is induced by renal and renovascular disorders. Congenital and vascular disorders, tumors, and history of renal disease may result in hypertension. Hypertension may lead to death, a sequel, or an irreversible damage in vital organs, such as heart, brain, and kidney, in children who do not undergo therapy despite early diagnosis (2-6).

The rising prevalence of overweight worldwide has led to an increased prevalence of essential hypertension among younger population. The present study was aimed at detecting the prevalence of hypertension and risk factors for hypertension among high school students in Sivas, Turkey.

## MATERIALS AND METHODS

This cross-sectional epidemiological study was conducted from 1 January to 31 March 2004. Using a multistage sampling method, six high schools, as clusters, were selected systematically from 26 high schools (with 14,789 students) in Sivas. In total, 1,041 students—from 9th to 11th grades were chosen randomly from those six high schools and examined for hypertension and some other risk factors that could possibly cause hypertension. The study group included 1,020 of targereted 1,041 students. In total, 1,020 students—593 males (58.1%) and 427 females (41.9%)—with the mean age of 15.9±1.0 (range 14–18) years were available for contact in the present study.

A questionnaire was used for obtaining information from students by self-reporting on age, gender, smoking, and whether they or their families have had any diseases, such as urinary tract infection and diabetes. The blood pressure, height, and weight of each student were measured by the research group. Other family members were contacted for diabetes or hypertension if the student had hypertension. Based on the present habit, the smoking status was evaluated for each student.

Measurements of arterial blood pressure were performed in a quiet room after five minutes of resting in a sitting position. Right arms were kept at the same level of heart during the measurement. Cuff bladder was arranged to cover about 75% of the upper arm, and the measurements were performed using a tool calibrated to cover the upper arm fully (7,8). The cuff was inflated until the radial pulse was no longer audible from the antecubital area, and then the cuff was deflated 2–3 mm Hg per second while auscultating the pulse. While decreasing the cuff pressure, the onset of the sound was systolic blood pressure of the student, and the disappearance of the sound was accepted as diastolic blood pressure. The measurements were performed three times repeatedly at an interval of five minutes by the same specialist medical doctors during 9 am–12 noon. First measurements were excluded, and the average of the last two measurements was taken into account. Two weeks later, measurements of blood pressure were repeated for students who were above the 95^th^ percentile according to the blood pressure percentile curves prepared by the USA Task Force Group. Students whose repeated systolic or diastolic blood pressures were higher than the 95^th^ percentile were considered to be hypertensive patients.

Body-weight and height were measured using digital scales (Soehnle, Germany) sensitive to 0.1 kg and 0.5 cm respectively, and a non-stretch tape was fixed to a flat vertical wall. Body mass index (BMI) was calculated dividing the weight by square of height (kg/m^2^). The BMI values that were considered to be underweight, overweight, and obesity were defined as <5th, ≥85th and ≥95th percentile, respectively, of age- and sex-specific National Center for Health Statistics/Centers for Disease Control and Prevention (NHCS/CDC 2000) (9).

Data obtained were analyzed using the SPSS software (version 10.0.5) (SPSS Inc., Chicago, IL, USA). Chi-square, Pearson correlation, and Student's *t*-test were used in evaluating data. Additionally, multiple linear regression analyses were performed. BMI, age, gender, smoking, presence of diabetes mellitus or urinary tract infection, and presence of diabetes mellitus or hypertension among the members of the family were included in the models as independent variables. Results were expressed as mean±standard deviation.

## RESULTS

Data of 1,020 students were used; 58.1% (n=593) of them were male, and 41.9% (n=427) were female. Their average age was 15.9±1.0 years. Table 1 shows the distribution of age-groups by gender.

**Table 1:** Distribution of age group by gender

Age (years)	Male (n=593, 58.1%)		Female (n=427, 41.9%)		Total (n=1020, 100.0%)	
	No.	%	No.	%	No.	%
14	28	46.7	32	53.3	60	5.9
15	173	56.2	135	43.8	308	30.2
16	194	55.6	155	44.4	349	34.2
17	156	64.5	86	35.5	242	23.7
18	42	68.9	19	31.1	61	6.0

4.4% (n=45) of the students had blood pressures above the 95^th^ percentile. Hypertension was prevalent among 5.4% of male and 3.0% of female students. Distribution of systolic and diastolic blood pressures by age and gender is given in Table 2. Both systolic and diastolic blood pressures were significantly (p<0.05) higher among the male students. Systolic blood pressures were higher among 18-year old male students, whereas diastolic blood pressures were significantly (p<0.05) higher than the values of female students in the 16–17 years old group.

**Table 2:** Distribution of systolic and diastolic blood pressures by age and gender

Age (years)	Systolic blood pressure (mean±SD)		Diastolic blood pressure (mean±SD)	
	Male	Female	Male	Female
14	109.6±9.4	111.9±9.0	70.5±9.4	73.6±7.5
15	111.2±10.3	109.3±9.1	71.3±8.6	70.1±8.4
16	112.4±10.6	110.0±9.0	72.7±8.4	70.7±7.6*
17	112.5±11.8	109.0±9.5	72.5±8.9	70.1±6.8*
18	111.7±10.7	108.7±6.8*	72.1±7.7	72.1±7.3
Total	111.9±10.7	109.7±9.0*	72.1±8.6	70.7±7.7*

*Student *t*-test, p<0.05

The male students had the mean height of 171.5±7.7 cm, the mean weight of 59.4±9.9 kg, and BMI of 20.1±2.6, whereas the female students had the mean height of 160.6±5.7 cm, the mean weight of 53.4±8.4 kg, and BMI of 20.7±2.9. All the three mean values were observed to be significantly (p<0.05) different with respect to gender.

Table 3 demonstrates the correlation of systolic and diastolic blood pressures with age, height, weight, and BMI. Although systolic and diastolic blood pressures did not significantly (p>0.05) correlate with age, statistically significant (p<0.05) positive correlation was observed with height, weight, and BMI.

**Table 3:** Relationship of systolic and diastolic blood pressures with age, height, weight, and body mass index in Pearson correlation analysis

Variable	Systolic blood pressure		Diastolic blood pressure	
	R	p value	R	p value
Age (years)	0.02	0.44	0.03	0.31
Height (m)	0.24	0.00	0.14	0.00
Weight (kg)	0.30	0.00	0.26	0.00
BMI (kg/m^2^)	0.26	0.00	0.23	0.00

Hypertension was detected in 5.9% of one or both parents, but could not be detected in 3.8% of parents of the hypertensive students. This difference was not statistically significant.

The involvement of some factors/variables in the prevalence of hypertension is shown in Table 4. The prevalence of hypertension was significantly higher among overweight students (18.4 %; p<0.05) than among non-obese students (4%). The figure shows the distribution of the prevalence of hypertension according to BMI. There was no significant difference between hypertension and age, gender, smoking, presence of diabetes mellitus, or hypertension among the members of the family. Multivariate regression analysis showed that only BMI was independently associated with hypertension (Table 5).

**Fig.**
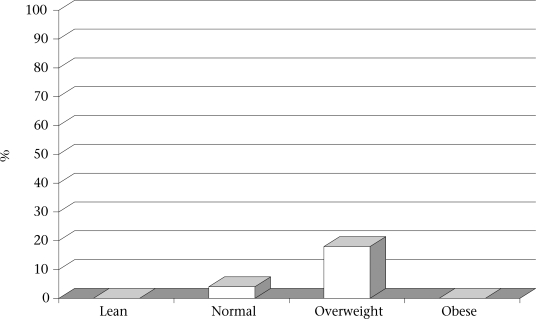
Prevalence of hypertension according to body mass index among study subjects

**Table 4:** Percentage of hypertension due to some variables in the present study

Variable	No.	%	Test*
Age (years)			
14	60	3.3	NS*
15	308	4.2	
16	349	4.9	
17	242	5.0	
18	61	1.6	
Gender			
Male	593	5.4	NS*
Female	427	3.0	
Smoking			
Yes	112	6.3	NS*
No	908	4.2	
Diabetes melitus			
Yes	6	-	NS*
No	969	4.4	
Family history of diabetes melitus			
Yes	160	3.8	NS*
No	860	4.5	
Family history of (hypertension)			
Yes	304	5.9	NS*
No	716	3.8	
Urinary tract infection			
Yes	110	7.3	NS*
No	910	4.1	
Body mass index			
Lean	20	-	0.00
Normal	962	4.0	
Overweight	36	18.4	
Obese	2	-	
Total	1,020	4.4	

*Chi-Square; NS=Not significant

**Table 5:** Relationship between hypertension and sociodemographic factors in multiple linear regression analysis

Independent variable	β	p value	95% confidence interval	
			Lower	Upper
Age (years)	0.08	0.60	−3.52	6.00
Gender	−0.23	0.16	−16.42	2.90
Smoking	0.22	0.19	−4.00	19.85
Family history of diabetes melitus	0.33	0.06	−0.86	26.75
Family history of hypertension	0.18	0.25	−3.63	13.6
Urinary tract infection	0.14	0.36	−9.06	24.2
Body mass index	0.50	0.00	0.62	3.43

Gender: Male=1, Female=2; Smoking, family history of diabetes melitus or hypertension and urinary tract infection: Yes=1, No=2

## DISCUSSION

In Turkish population, the prevalence of hypertension was 5.8% in the age-group of 13–20 years (10), 7.2% in the age-group of 13–18 years (11), 4.8% among females and 3.8% among males of 7–18 years age-group (12). The prevalence of hypertension was 6.9% in the age-group of 6–18 years in Brazil (13), 6.0% in the age-group of 14–19 years in the United States (14,15), 5.1% in the age-group of 8–16 years in Papua New Guinea (16), 2.9% in the age-group of 4–18 years in Germany (17), and 3.4% in the age-group of 4–18 years in Argentina (18). In our study, the prevalence of hypertension was 4.4%. The prevalence of hypertension reported in this study is consistent with the prevalence reported in the literature. The reason for the difference between the prevalence of hypertension in this study and those in the previous studies might be the usage of different age-groups.

In the present study, blood pressure correlated positively with height, weight, and BMI significantly (p=0.00). Furthermore, the BMI was also significantly (p=0.00) associated with hypertension after multivariate regression analysis. This result is consistent with the results of previous studies (19-22). As reported in some studies, race and ethnicity also have an impact on blood pressure. Blood pressure values are particularly higher for black and south East Asian races (23,24).

According to Turkish literature, investigating the association among age-groups, gender, and mean blood pressure values, Kıyak *et al*. reported the higher mean systolic and diastolic blood pressure values for girls aged 6–11 years when compared with their male peers (20). Tümerdem *et al*. stated that the mean systolic and diastolic blood pressure values for boys aged 7–12 years were higher than those of girls (21). Özkan *et al*. reported the higher mean diastolic blood pressure for girls aged 7–12 years (22) and the higher mean systolic blood pressure for boys aged 7–8 years and for girls aged 9–12 years. The study by Cşokun *et al*. demonstrated the similar mean systolic blood pressure values for both genders between the ages of 7 and 10 years (25). However, the mean systolic blood pressure was higher for boys aged 11–15 years, and the mean diastolic blood pressure values were higher for girls in both the age-groups. In a study by Koç *et al*., the mean systolic blood pressure values for boys at the ages of 6 and 15 years were observed to be higher, while girls had higher values in other age-groups (26). On the other hand, there were no significant gender-related differences in terms of the mean diastolic blood pressure values. According to data obtained in the present study, while the mean values for systolic blood pressure were higher for boys aged 18 years, the mean diastolic blood pressure values were significantly higher for boys aged 16–17 years. Boys in general had significantly higher mean values for both systolic and diastolic blood pressures when all the subjects enrolled were considered. On the other hand, when the blood pressure values were compared with respect to age-groups for both genders, no significant differences were observed. The discrepancies between studies may be attributed to socioeconomic and environmental factors.

If any one of the parents is hypertensive, the probability of being hypertensive for their children may be 28%. If both the parents are hypertensive, the probability of being hypertensive for their children may be 41.0% (27). Our results showed no significant difference between parents with and without hypertension.

It is known that infections in the urinary system, particularly reflu-dependent nephropathy and parenchyma damage, may lead to hypertension (28,29). In this study, the prevalence of hypertension was 7.3% among students who had urinary infection in the past and 4.1% among the non-infected group. There was no significant difference between these groups. It might be that the discrepancy might be based on memory factor.

Hypertension was demonstrated as an important public-health problem among high school students in Sivas. Our results showed that BMI was an important parameter in hypertension in such a study group. Frequent measurement of blood pressure of such cases, especially students with overweight, could be an effective preventive method for the early detection of hypertension and its complications. Effective therapy and continuous health education concerning healthy lifestyle are also essential in this age-group.
